# Effect of Vitamin D Supplementation on Glycemic Control in Newly Diagnosed Patients With Type 2 Diabetes Mellitus: A Randomized Controlled Trial

**DOI:** 10.7759/cureus.70224

**Published:** 2024-09-26

**Authors:** Kuldeep Kumar, Tidip Bordoloi, Shiva Narang, Mrinalini Kotru, Aditi Singh

**Affiliations:** 1 Department of Medicine, University College of Medical Sciences, Guru Teg Bahadur Hospital, New Delhi, IND; 2 Department of Pathology, University College of Medical Sciences, Guru Teg Bahadur Hospital, New Delhi, IND

**Keywords:** hba1c, homa-ir, insulin resistance, type 2 diabetes, vitamin d

## Abstract

Objective: Vitamin D deficiency is commonly associated with type 2 diabetes and it has been linked to impaired glycemic control in these patients. This study was done to determine if vitamin D supplementation improves glycemic parameters in type 2 diabetes.

Methods: This randomized controlled trial was done in 140 newly diagnosed adult patients with type 2 diabetes. The participants were randomly divided into two groups where one group received vitamin D (60,000 International Units (IU) of oral vitamin D3 weekly for a duration of three months, followed by 1000 IU twice daily for another three months in vitamin D deficient patients; 1000 IU (25 mcg) twice daily for six months for subjects with sufficient vitamin D level) along with standard anti-diabetic drugs while the other group received standard anti-diabetic drugs only. The effect of vitamin D supplementation was assessed by measuring glycated haemoglobin (HbA1c), fasting blood sugar (FBS), postprandial blood sugar (PPBS), fasting insulin, and insulin resistance as Homeostasis Model Assessment of Insulin Resistance (HOMA-IR) at the baseline and after six months.

Results: Vitamin D deficiency was observed in 60% (n=84) of the subjects with the rest 40% (n=56) having sufficient serum vitamin D levels. The baseline mean HbA1c was 8.48 ± 1.46% and 8.21 ± 1.24% in the control and vitamin D group, respectively. After supplementing vitamin D for six months, no significant difference was observed between the two groups in terms of HbA1c (p= 0.263). Similarly, there was no significant change in other parameters like FBS, PPBS, fasting insulin, and insulin resistance (HOMA-IR). A subgroup analysis within the vitamin D group between the vitamin D sufficient and deficient patients also revealed no significant changes in the above parameters.

Conclusion: Vitamin D supplementation, within the parameters of this study, did not yield a distinctive advantage in improving glycemic outcomes in individuals with type 2 diabetes.

## Introduction

Type 2 diabetes mellitus is a global health concern characterized by insulin resistance and impaired insulin secretion. The prevalence of type 2 diabetes mellitus has reached epidemic proportions, with millions of individuals affected worldwide. While genetic and lifestyle factors contribute to the development of type 2 diabetes mellitus [1], emerging research suggests a potential link between vitamin D deficiency and the risk of developing this metabolic disorder [2].

Vitamin D is the major steroid hormone involved in the regulation of mineral ion homeostasis. Over the past decade, it has garnered significant attention for its extracellular roles in various conditions, including diabetes mellitus. Insulin resistance, a key characteristic of type 2 diabetes mellitus, has been linked to vitamin D, as studies suggest its involvement in regulating insulin sensitivity. The presence of vitamin D receptors (VDR) in insulin-sensitive tissues indicates its potential impact on insulin action through intracellular signalling pathways. Additionally, experimental evidence indicates that vitamin D can influence the expression of genes involved in insulin secretion and glucose metabolism [3]. Chronic low-grade inflammation contributes to the development of insulin resistance [4], and vitamin D's anti-inflammatory properties may help alleviate inflammation associated with type 2 diabetes mellitus [5]. Moreover, vitamin D plays a role in immune regulation, and its deficiency has been associated with autoimmune processes that could contribute to dysfunction in pancreatic beta cells [6]. Adipose tissue, crucial for energy balance and insulin sensitivity, contains VDR. Vitamin D may affect adipokine secretion, thereby influencing insulin sensitivity [7]. Studies suggest that vitamin D can modulate adiponectin, an adipokine known for its insulin-sensitizing properties, potentially leading to improved metabolic outcomes [7]. These findings highlight the multifaceted role of vitamin D in the pathophysiology of type 2 diabetes mellitus, extending beyond its classical functions in mineral ion regulation.

Clinical trials assessing the impact of vitamin D supplementation on glycemic control and insulin sensitivity have shown mixed outcomes. Factors such as baseline vitamin D status, dosage regimens, and duration of supplementation contribute to the variability in results. Also, various trials have explored the benefits of vitamin D supplementation on glycemic control in patients, but very few of them combined vitamin D supplementation with standard anti-diabetic drugs. Hence, we designed this study to know about the effects of vitamin D supplementation along with standard anti-diabetic drugs on various glycemic parameters in patients with type 2 diabetes mellitus.

## Materials and methods

Aim

To study the effect of vitamin D supplementation on glycemic control in newly diagnosed type 2 diabetes mellitus patients.

Primary objective

1. To study the effect of vitamin D supplementation on glycated haemoglobin (HbA1c) after six months.

Secondary objective

1. To know vitamin D status in newly diagnosed type 2 diabetes mellitus patients.

2. To study the effect of vitamin D supplementation on insulin resistance after six months.

Study design and participants

The study was a single-blind, prospective randomized controlled trial (RCT). It was conducted in the ward and outpatient department (OPD) of the Medicine Department and the Diabetes, Endocrinology, and Metabolism Department of Guru Teg Bahadur Hospital, New Delhi, India. The participants were recruited between September 2022 and February 2024. The trial was registered in Clinical Trials Registry- India (CTRI reg. no.: CTRI/2022/10/046633, reference no.: REF/2022/08/057502). Institutional ethical clearance was also obtained.

Adult subjects (18-60 years) of recently diagnosed type 2 diabetes mellitus who were attending the Medicine or Endocrine OPD were screened for participation in our study and those who gave consent were included in our study. The main exclusion criteria were as follows: any patients with type 1 diabetes mellitus patient or type 2 diabetes mellitus patient who were already on oral hypoglycemics or insulin, an impaired renal function (estimated glomerular filtration rate (eGFR) <30 mL/min/1.73 m^2^, calculated from serum creatinine using the Modification of Diet in Renal Disease formula), hypercalcemia (serum calcium >2.65 mmol/L), severe anaemia, chronic liver disease, alcoholism, pregnancy and any patient who was already on vitamin D supplements.

Intervention

**Figure 1:**
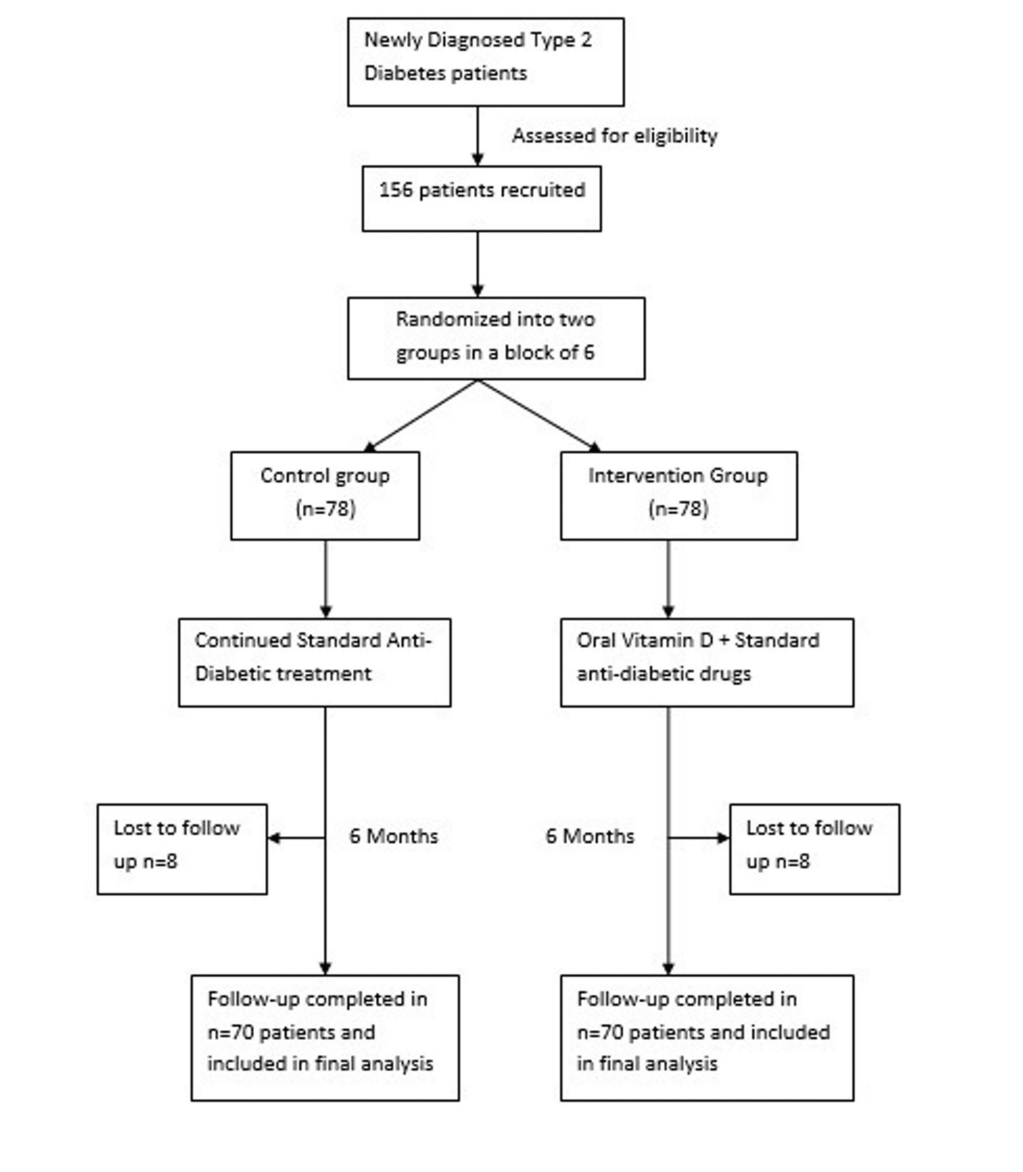
Participant flowchart

The participants recruited were randomly divided into two groups. The subjects were randomized by stratified block randomization in a block of six so that an equal number of subjects fell into each group. In one group, the patients were prescribed oral vitamin D3 therapy and continued their prescribed drug therapy for type 2 diabetes for six months, while the other group continued their drug therapy for type 2 diabetes only. The vitamin D deficient participants in the first group (defined as having serum levels of 25-hydroxyvitamin D (25(OH)D) ≤20 ng/mL) [8] were asked to take 60,000 International Units (IU) of oral vitamin D3 weekly for three months period, followed by 1000 IU twice daily for another three months. The participants whose 25(OH)D level was >20 ng/mL were asked to take 1000 IU (25 mcg) twice daily until the end of the study i.e., for six months (Figure 1).

Outcome measures

The primary outcome of the trial was the change in serum HbA1c level between the vitamin D plus standard drug group and the standard drug-only group after six months of intervention. The secondary outcomes were to know the status of vitamin D in newly diagnosed type 2 diabetes mellitus and the change in insulin resistance level in both the groups (measured by Homeostasis Model Assessment of Insulin Resistance (HOMA-IR)) after six months of intervention.

Statistical analysis

We calculated that 140 patients with type 2 diabetes would be required in this trial to demonstrate a significant difference at 80% power and 5% significance. Power calculations were based on previously published literature. According to an expected drop-out rate of 10%, 156 subjects were recruited.

The data was entered in a Microsoft Excel spreadsheet (Microsoft® Corp., Redmond, USA) and analysis was done using Epi-Info (Centers for Disease Control and Prevention (CDC), Atlanta, USA), JASP (Jeffreys's Amazing Statistics Program; University of Amsterdam, Netherlands), and Statistical Package for the Social Sciences (IBM SPSS Statistics for Windows, IBM Corp., Version 25.0, Armonk, NY). Continuous variables are represented as mean ± SD or medians with an interquartile range. Categorical variables are represented as numbers and percentages (%). All tests of significance were two-tailed and statistical significance was defined as p < 0.05. Appropriate non-parametric tests (i.e., Mann-Whitney test) were used for all analyses for statistical significance.

## Results

The primary objective of our study was to assess whether vitamin D supplementation improved glycemic control in patients who were newly diagnosed with type 2 diabetes. We screened all patients of recently diagnosed type 2 diabetes mellitus who were attending the Medicine or Endocrine OPD for participation in our study. After the application of appropriate inclusion and exclusion criteria, we were able to successfully recruit and complete the follow-up in a total of 140 subjects for our study. The baseline demographic, biochemical, and anthropometric characteristics of both groups are presented in Table 1. Most of our subjects (58%) were above 50 years old with a median age of 45.0 years. There was a slight female preponderance with females being 51.43% (N=72). The majority belongs to the overweight (12.14%, N=17) and obese (87.14%, N=122) category. Our subjects displayed uncontrolled glycemic parameters, with the vast majority experiencing uncontrolled fasting (99.29%, N=139) and postprandial blood sugar (PPBS) (97.86%, N=137) levels, along with elevated HbA1c (87.13%) levels. The mean HbA1c was 8.34 ± 1.36%. Among the subjects, 60% (N=84) were observed to have insufficient levels of vitamin D (below 20 ng/mL). Overall mean serum 25(OH)D was 16.34 ± 7.91 ng/mL. Both groups were found to be comparable at baseline in terms of glycemic parameters.

**Table 1:** Patient demographics and baseline characteristics Data are presented as mean ± SD, unless indicated otherwise. HbA1c: glycated haemoglobin; HOMA-IR: Homeostasis Model Assessment of Insulin Resistance; 25(OH)D: 25-hydroxyvitamin D

	Control Group (N=70)	Vitamin D Group (N=70)
Demographic Parameters		
Male, N (%)	33 (47.14%)	35 (50.0%)
Age (years)	44 ± 9.0	45.6 ± 9.76
Clinical Characteristics		
BMI (kg/m^2^)	28.5 ± 2.4	27.82 ± 2.6
Fasting glucose (mg/dL)	213.3 ± 65.93	203.87 ± 66.54
Postprandial blood glucose (mg/dL)	279.19 ± 81.56	266.27 ± 72.85
HbA1c	8.48 ± 1.45	8.21 ± 1.23
Fasting insulin (mIU/mL)	13.7 ± 8.35	13.34 ± 5.44
HOMA-IR	6.83 ± 4.33	6.32 ± 2.19
Serum 25(OH)D (ng/mL)	15.23 ± 7.67	17.46 ± 7.94
Vitamin D deficiency, N(%)	45 (64.3%)	39 (55.7%)

Regarding the primary outcome, the change in HbA1c from baseline to six months did not differ significantly between both groups (median reduction control group: 0.5 g/dL, vitamin D group: 0.7 g/dL, p-value = 0.263). Regarding the change in the secondary outcomes, both groups exhibited similar changes in the levels of fasting blood sugar (FBS) (median reduction control group: 43.0 mg/dL, vitamin D group: 43.0 mg/dL, p-value = 0.734), PPBS (median reduction control group: 45.0 mg/dL, vitamin D group: 59.0 mg/dL, p-value = 0.147), fasting insulin (median increase control group: 2.06 uIU/mL, vitamin D group: 2.44 uIU/mL, p-value = 0.236) as well as HOMA-IR (median reduction control group: 0.53, vitamin D group: 0.45, p-value = 0.899). The same has been shown in Table 2.

**Table 2:** Comparison of characteristics before and after treatment in both groups Data are presented as median (IQR), unless indicated otherwise. The p-value is considered significant if p<0.05. HbA1c: glycated haemoglobin; HOMA-IR: Homeostasis Model Assessment of Insulin Resistance

	Control Group (n=70)		Vitamin D Group (n=70)		
	0 months	6 months	0 months	6 months	p-value
A: Entire Study Population					
Fasting glucose(mg/dL)	195.5 (166.0-241.0)	157.0 (136.75-186.0)	192.0 (157.0-230.75)	153.0 (126.5-175.75)	0.734
Postprandial blood glucose (mg/dL)	253.5 (221.5-301.0)	209.5 (191.0-236.75)	256.0 (212.0-290.75)	202.5 (172.0-225.0)	0.147
HbA1c (%)	7.95 (7.4-9.38)	7.4 (7.12-8.3)	8.0 (7.12-8.88)	7.3 (6.8-7.7)	0.263
Fasting insulin (mIU/mL)	12.71 (9.01-15.6)	13.56 (11.19-18.32)	12.15 (9.4-15.38)	15.04 (11.34-19.11)	0.236
HOMA-IR	6.09 (4.93-7.3)	5.38 (4.64-6.9)	6.1 (4.65-6.89)	5.4 (4.42-6.85)	0.899

A subgroup analysis was conducted within the vitamin D group between the vitamin D deficient and sufficient subjects to compare whether there were any differences in glycemic parameters with respect to the baseline vitamin D status. In the vitamin D group, 39 (55.7%) subjects had deficient vitamin D levels (<20 ng/mL) while 31 (44.3%) subjects had sufficient vitamin D levels. Analysis between the above two groups also revealed no significant changes in the levels of FBS (median reduction deficient group: 39.0 mg/dL, sufficient group: 43.0 mg/dL, p-value = 0.558), PPBS (median reduction deficient group: 62.0 mg/dL, sufficient group: 54.0 mg/dL, p-value = 0.149), HbA1c (median reduction deficient group: 0.7%, intervention group: 0.7%, p-value = 0.877), fasting insulin (median increase deficient group: 2.71 uIU/mL, sufficient group: 2.14 uIU/mL, p-value = 0.864) as well as HOMA-IR (median reduction deficient group: 0.35, sufficient group: 0.69, p-value = 0.056). The same has been shown in Table 3.

**Table 3:** Comparison of characteristics after treatment in the intervention group between vitamin D deficient and sufficient subjects Data are presented as median (IQR), unless indicated otherwise. The p-value is considered significant if p<0.05. HbA1c: glycated haemoglobin; HOMA-IR: Homeostasis Model Assessment of Insulin Resistance; FBS: fasting blood sugar; PPBS: postprandial blood sugar

Change in Parameters (Baseline - After Six Months)	Intervention Deficient	Intervention Sufficient	p-value
FBS (mg/dL)	39 (30-54)	43 (35-57)	0.558
PPBS (mg/dL)	62 (45-86)	54 (36-69)	0.149
HbA1c	0.7 (0.4-1.6)	0.7 (0.5-1)	0.877
Fasting Insulin (uIU/mL)	-2.71 (-4.22 to -0.66)	-2.14 (-4.08 to -1.17)	0.864
HOMA-IR	0.35 (0.09-0.75)	0.69 (0.18-1.12)	0.056

## Discussion

The primary objective of this RCT was to investigate the potential benefits of vitamin D supplementation on glycemic control in individuals with diabetes. Our primary focus was to compare the degree of improvement between the control and vitamin D groups. Calculating changes in values after six months, we found similar improvements in FBS, PPBS, HbA1c, fasting insulin, and HOMA-IR between the groups. The lack of statistically significant differences in these parameters suggests that vitamin D supplementation did not lead to a superior improvement in glycemic control compared to the control group.

A large proportion of our study subjects (60%) were found to be vitamin D deficient with levels falling below the threshold of 20 ng/mL. This is consistent with findings from another Indian study conducted by Dutta et al. [9] who reported vitamin D deficiency in 73.52% of participants in their study on prediabetics (125 out of 170 participants). Our findings on the effect of vitamin D on various glycemic parameters were also consistent with various other studies. An RCT by Dutta et al. [9] on prediabetics also yielded similar findings, where they reported no significant decrease in insulin resistance (HOMA-IR) between the vitamin D supplementation group vs. the placebo group. Further RCTs by Elkassaby et al. [10] and Krul-Poel et al. [11] also had similar findings with no significant change in FBS, PPBS, HbA1c, and insulin resistance (HOMA-IR) levels between the intervention and placebo groups after six months of vitamin D supplementation.

Our study had important limitations. Firstly, the study's relatively small sample size of 140 subjects from a single centre might limit the generalizability of findings to a broader population and also potentially introduce bias related to geographical and demographic factors. Second, the study did not employ a double-blinded design. The lack of blinding could influence the assessment of outcomes and introduce unintended biases, impacting the internal validity of the study. Thirdly, the dosage of vitamin D used for supplementation was not based on empirical data. Since we did not evaluate the levels of vitamin D after six months, the effectiveness of our vitamin D supplementation in the patients could not be studied. Lastly, the absence of a placebo-controlled arm in the study design limits the ability to differentiate the specific effects of vitamin D supplementation from potential placebo effects.

## Conclusions

In conclusion, while our study contributes valuable data to the ongoing discourse on vitamin D and glycemic control, the overall evidence suggests that vitamin D supplementation, within the parameters of this study, does not yield a distinctive advantage in improving glycemic outcomes in individuals with diabetes. Further exploration and large-scale studies are needed to corroborate our findings. However, given the considerable proportion of patients in our study and others with vitamin D deficiency, it may be prudent to include an assessment of serum vitamin D levels upon diagnosis of type 2 diabetes, considering its potential skeletal benefits.
